# The Gliocentric Brain

**DOI:** 10.3390/ijms19103033

**Published:** 2018-10-05

**Authors:** James M. Robertson

**Affiliations:** 508 27th Avenue South, North Myrtle Beach, SC 29582, USA; jimrobertsonmd@yahoo.com

**Keywords:** astrocytes, neuro-glial interactions, neurodegenerative disease, memory, consciousness, behavior

## Abstract

The Neuron Doctrine, the cornerstone of research on normal and abnormal brain functions for over a century, has failed to discern the basis of complex cognitive functions. The location and mechanisms of memory storage and recall, consciousness, and learning, remain enigmatic. The purpose of this article is to critically review the Neuron Doctrine in light of empirical data over the past three decades. Similarly, the central role of the synapse and associated neural networks, as well as ancillary hypotheses, such as gamma synchrony and cortical minicolumns, are critically examined. It is concluded that each is fundamentally flawed and that, over the past three decades, the study of non-neuronal cells, particularly astrocytes, has shown that virtually all functions ascribed to neurons are largely the result of direct or indirect actions of glia continuously interacting with neurons and neural networks. Recognition of non-neural cells in higher brain functions is extremely important. The strict adherence of purely neurocentric ideas, deeply ingrained in the great majority of neuroscientists, remains a detriment to understanding normal and abnormal brain functions. By broadening brain information processing beyond neurons, progress in understanding higher level brain functions, as well as neurodegenerative and neurodevelopmental disorders, will progress beyond the impasse that has been evident for decades.

## 1. The Neuron Doctrine

“To pursue mind into the unicellular would seem like looking for a firefly across astronomical distances.” Sir Charles Sherrington [1].

The Neuron Doctrine states that the neuron is the elemental unit underlying all cognitive functions. Additionally, it is considered autonomous developmentally, metabolically, and independent of trophic influences from other neurons or glia.

The quintessential neuron is familiar to all neuroscientists. Dendrites receive information, via action potentials, that is integrated within the soma and subsequently propagated along axons to signal dendrites on other neurons across a discontinuous space, designated the synapse. The term dynamic polarization was coined to represent this unidirectional transfer of information.

### 1.1. Axodendritic Synapse

The discontinuity of synapses was confirmed by electron microscopy [2]. Additional ultrastructural studies subsequently documented multiple exceptions to the classical axo-dendritic synapse. This includes “axo-axonic, dendro-dendritic, somato-somatic, somato-dendritic, dendro-somatic, dendro-axonic and somato-axonic synapses” [3].

Most remarkable was the discovery of synapses on astrocytes in the visual cortex and oligodendrocytes [4,5]. Synapses also occur on oligodendrocyte precursor cells throughout the adult frontal neocortex [6,7]. Axo-glial synapses are an obvious contradiction of the Neuron Doctrine.

### 1.2. Dynamic Polarization

The discovery of nonsynaptic communication between neurons is the most damaging evidence contradicting the Neuron Doctrine. Electrical synapses are formed by gap junction connections between dendrites of adjoining inhibitory neurons. Not only are they incompatible with the principle of dynamic polarization, but are more compatible with a reticular interpretation, since there is cytoplasmic continuity between cells [8].

Electrical synapses are crucial to normal cognitive functions. Their “rapid response is critical to inhibitory control of primarily glutamatergic excitatory neurons”. They also “contribute to oscillatory rhythms observed in electroencephalography (EEG) and magnetoencephalography (MEG) recordings” [9]. This includes gamma oscillations that are important in the timing of perceptual elements prior to consciousness expression and memory formation [10].

Furthermore, action potentials into pyramidal cells have been shown to “back propagate into dendrites” which have 5000–50,000 “post synaptic receiving sites” [9]. This is contradictory to the unidirectional signaling proposed in the Neuron Doctrine. Retrograde messengers, such as nitrous oxide, are also important non-synaptic factors that contribute to synaptic activation.

### 1.3. Developmental Autonomy

The original concept that neurons originate from dedicated embryonic neuroepithelial cells (i.e., neuroblast of His) is invalid [11,12,13,14]. “Radial glia cells (RGCs) are embryonic pluripotent precursors that direct mammalian neurogenesis and gliogenesis in a highly ordered chronological fashion” [15]. Additionally, astrocytes in the subgranular zone of the adult hippocampus and subventricular region maintain the ability to undergo neurogenesis [16,17], in contradiction to the Neuron Doctrine that states that the number and placement of neurons in the adult neocortex are fixed.

### 1.4. Metabolic Independence

It has long been known that neurons are incapable of fully supplying their metabolic needs. Neuronal activity shuts down when astrocyte-specific metabolic inhibitors are utilized. Furthermore, such inhibitors disrupt memory formation as well [18].

Astrocytes provide lactate as an essential energy source to neurons [19]. It has been shown that “the supply of energy is activity dependent, with lactate or its precursor glucose being able to travel through gap-junctions between astrocytes to reach the location where it is required” for synaptic transmission [20]. It is also essential for the maintenance of hippocampal long term potentiation (LTP), which is assumed essential for memory formation [21].

Cholesterol, in particular, provides building material for new membranes that appear during synaptogenesis [22]. It is transported from the vascular compartment by astrocytes to ultimately provide this critical molecule to neurons. Interestingly, the cholesterol is bound to APOE, which is a genetic marker for increased risk of developing Alzheimer’s disease.

Astrocytes are the only source for the synthesis of glutamate from glucose in the neocortex. Glutamate, the major excitatory neurotransmitter, cannot be synthesized in neurons because they lack glutamine synthetase as well as pyruvate decarboxylase. They are expressed in astrocytes that ultimately supply glutamine to neurons, which can be further synthesized into glutamate and/or GABA. This is known as the glutamine-glutamate (GABA) shuttle, “which is indispensable for maintenance of excitatory and inhibitory neurotransmission” [23].

The rapid uptake of glutamate during synaptic activity is critical for neuronal survival and normal function, since the large amount of glutamate discharged into the synaptic cleft is excitotoxic if not rapidly removed. Astrocyte glutamate and GABA transporters are essential for rapid removal of these neurotransmitters from synapses [24,25].

### 1.5. Trophic Isolation

The independence of neurons from glia and other cells that produce and release trophic molecules is another significant error of the Neuron Doctrine. This is particularly true during synaptogenesis [26]. Numerous astrocyte-secreted molecules have been identified that are chronologically expressed to manifest the number as well as the morphological and physiological integrity of synapses during development and into adulthood [27]. Over twenty cell adhesion molecules and matricellular protein trophic factors that support the development and maintenance of synapse have been identified so far [28,29].

Peripheral astrocyte processes (PAP) are associated with synapses. Their terminals are predominately located around postsynaptic sites. Astrocyte Connexin 30 (Cx30) acts as an adhesive molecule to confine the PAP to this area [30]. “Functionally, astrocytic Cx30 regulates long-term synaptic plasticity and hippocampal-based contextual memory” [31].

### 1.6. Cognition

The central role of the synapse in cognition was proposed almost simultaneously with the Neuron Doctrine when studies involving spinal reflex arcs exhibited both excitatory and inhibitory synaptic components. Sherrington noted a paradox when extending these findings to cognitive functions: “But when trying to colocate nerve action with mental activity we face something which not only transmits signals but reads them”. He concluded: “The mental action lies buried in the brain, and in that part most deeply recessed from the outside world, that is furthest from input and output” [1]. Although he had no way to know, the “most deeply recessed” region beyond the synapse is the location of the astrocyte syncytium (AS).

The AS is a globally distributed complex network of astrocytes that are interconnected by gap junctions composed of Cx30 and Cx43 [32,33,34,35,36,37]. Connexins are protein subunits that collectively form gap junctions. Six connexins form one-half of a gap junction and are located in the membrane of one cell. This connects to six connexins on an adjoining cell to complete the formation of a gap junction that allows intercellular transfer of molecules that are essential for tissue homeostasis. Uniquely, the syncytium consists of individual astrocytes that form non-overlapping domains that are contiguous and continuous [38,39,40]. Therefore, the domains of the AS tile the entire neocortex in a three-dimensional geometric pattern [15].

Importantly, the interconnecting gap junctions between astrocyte domains allow the distribution of small molecules, many important in information processing and metabolic homeostasis, throughout the AS. Such molecules are relatively small (up to 1 kilodalton) and include glutamate, glucose, lactate, ATP, cyclicAMP, IP3, cytokines, calcium and other ions, small inhibitory RNA, micro RNA, and other small molecules [15,23,34,35,36,37,41].

Three decades of accumulating empirical data demonstrates that astrocytes are critical for complex information processing. Therefore, many gliobiologist conclude that these cells are crucial for cognitive functions, including learning and behavior [15,42,43,44,45,46,47,48,49].

Each human astrocyte within its domain is characterized by long peripheral processes that constitute 85% of the cell surface and is associated with as many as two million synapses [39,40]. Such perisynaptic processes (PSP) contain the vast majority of astrocyte receptors, transporters, and gap junctions [15,46,47,49]. Astrocyte synaptogenic molecules are also released from this region [27,50]. The term tripartite synapse has been proposed to indicate the importance of astrocytes as equal partners with neuronal presynaptic and postsynaptic terminals in synaptic information processing.

The characteristics of the AS led to the radical proposal that the AS is the ultimate site for the expression of cognitive functions, “where the thoughts dwell” [51], including consciousness [15,44,52,53,54,55] and memory formation [15,52,56,57]. Therefore, the synapse is considered the penultimate site of information processing [52].

“Astroglial networks may represent a wiring system parallel to that formed by neurons. The gap junctional connectivity may also represent a specialized ‘analog’ intercellular signaling system (alternative to ‘binary’ interneuronal communication), which by utilizing intercellular diffusion of multiple molecules, can provide a second level of information processing in the CNS” [23]. Interestingly, such a binary/analog hybrid system was also proposed by Dennett [58] as characteristic of consciousness.

Pereira and Furlan [54] propose that the role of global gamma synchrony is to “induce the transfer of information patterns embodied in local field potentials to astrocytic calcium waves” to express consciousness and are further “responsible for the ‘binding’ of spatially distributed patterns into unitary conscious episodes”.

The term “binding” may be a misnomer. It has been proposed that astrocytes gap junctions are the locus of conscious representation. Quintillions of gap junctions are primarily located in PSP terminals. Perception is projected by synapses onto these highly ordered structures that exist in tessellating hexagonal arrays that coalesce to form gap junctional plaques. They are associated with molecules known to be involved in cognitive functions [59].

Uniquely, astrocyte gap junctions exist in a quasicrystalline form that has the ability to crystalize [60]. Consciousness is postulated to occur when gap junctions are activated by synaptic activity. Memories are formed when gap junctions convert to a crystalline configuration. The type of explicit memory (iconic, short term, long term) depends on the time interval between the original conscious moment and return to the quasicrystalline state. Reconsolidation of the memory occurs with the return to the crystalline state after recalling a specific memory [15,52].

This hypothesis is extraordinarily parsimonious. It is the only one that the author is aware of that proposes a mechanism for memory reconsolidation. Metaphorically, consciousness represents the movie screen of an analog movie projector (which projects visual and auditory perceptions sequentially as the film frames advance).

However, the proposal is immensely more complex. Astrocytic gap junctions represent quintillions of “pixels” on the hypothetical screen, resulting in an enormous depth of visual acuity. Furthermore, because astrocyte gap junctions are heavily localized in PSPs to form a three-dimensional global network throughout the brain, the result is not a two-dimensional screen but an extremely complex tessellating three-dimensional display. This is how consciousness is a priori experienced within the brain [15].

Another hypothesis of consciousness utilizing a projector metaphor was introduced by Crick and Koch [61]. Known as the Snapshot Hypothesis, their proposal was based on the phenomenon of “cinematographic vision”, a manifestation of visual migraines, in which the sufferer experiences “a succession of ‘stills’, without any movement between the images, like the flickering of a movie run too slowly”.

### 1.7. Empirical Evidence Supporting a Role for Astrocytes in Cognitive Functions

1. “Astrocytes are capable of expressing virtually any type of receptor found in the CNS, which allows astroglia to perceive the neurochemical landscape of the nervous tissue” [23].
2. Astrocyte receptors can distinguish signals from neuronal pathways releasing the same neurotransmitter (e.g., glutamate) or different neurotransmitters (e.g., acetylcholine and glutamate) from diverse origins [62,63,64]. They respond with two types of intense increases in intracellular calcium concentrations that are “clearly separable because of their distinct temporal characteristics” [65]. The first is extremely rapid (98 ms) with an “immediate interaction between neurons and astrocytes” [65]. The other is characterized by a subsequent slow rise in calcium levels. Therefore, astrocytes and other glia are “calcium excitable” compared to “electrically excitable” neurons [62,66,67,68,69,70,71,72,73,74,75,76,77,78,79,80,81]. Astrocyte calcium waves occur with increased intensity of stimuli. In such cases, the calcium spreads in a wave-like fashion via gap junctions through the AS to affect multiple surrounding astrocyte domains.
3. The rapid detection of synaptic activity is the same in astrocytes as neurons [65,78]. This is the time frame in which consciousness is expressed [61]. The slower time scale is more compatible with working memory [82] or the beginning of longer term memory formation.
4. Astrocytes express, store, and consolidate information from individual synapse to global neuronal networks [15,23,27,44,46,49,51,83,84].
5. Human astrocytes have undergone dramatic evolutionary exaptations. This is in contrast to neurons, whose cell types, electrical properties, and depth of lamination have remained relatively constant in spite of large differences in overall size compared to other mammals [85,86,87]. This includes a magnocellular exaptation of protoplasmic astrocytes in primates which are 27-fold larger than rodent astrocytes. Three primate-specific astrocytes have been described (interlaminar, polarized, and varicose projection astrocytes) [39,40,88]. The complexity of the intralaminar astrocytes correlates with the relative intelligence observed between groups of primates [89].
  Importantly, since there has been virtually no change in the depth or cellularity of the neocortex in mammals, the volume of primate magnocellular protoplasmic and exapted astrocytes represent a huge presence within the human neocortex relative to non-primate brains. For instance, the volume should be much greater than the volume of astrocytes in the adult rat brain, which accounts for 50% of the volume of the neocortex in this species [90].
6. It has been proposed that “enormous pressures developed for alternatives to information processing and cognition as brains enlarged and neurons became fastidious in order to maintain the rapid transfer of information encoded in action potentials over ever-increasing distances to relatively fewer neurons” [48]. For instance, the adult mouse brain is estimated to have 142,000 neurons per cubic millimeter, whereas elephants have 6000 per cubic millimeter [87]. A logical conclusion is that the enormously enlarged human protoplasm astrocytes and their exapted primate and human-specific cohorts assumed sentient functions [48].
7. Interlaminar astrocytes have received particular attention because of their location, and empirical findings indicating a role in increased learning. These cells are located in Lamina 1 and send the majority of their protoplasmic processes within this lamina and fewer down to Lamina 4 and up to the glia limitans [39,40].

Lamina 1 consists primarily of synapses, particularly the apical dendrites of cortical pyramidal neurons. Eccles [91] concluded that this is the location in which cognitive processes are likely to manifest because of the confluence of thalamocortical and cortico-cortical synapses that terminate on pyramidal neuron apical dendrites in this region. Importantly, pyramidal neurons constitute 70% of the cells in the neocortex [92].

Han et al. [93] and associates research supports the proposal that interlaminar and human protoplasmic astrocytes are associated with cognitive functions. Xerographic human astrocyte progenitor cells transplanted into immune suppressed neonatal mice results in a preponderance of human protoplasmic and intralaminar astrocytes when the mice mature. Remarkably, the chimeric mice exhibit extremely rapid learning of three hippocampal-related memory test and fear conditioning compared to normal mice. This supports the idea that the evolutionary exaptation of primate-specific astrocytes are “important players in the improvement of cognitive abilities during human evolution” [39].

## 2. The Primary Role of Synapses in Cognitive Functions

“But how the heck do these synapses retain a stable identity when the chemistry of cells is almost on the boil, with large molecules falling apart nearly as soon as they are made?” McCrone [94].

LeDoux [95] succinctly states the contemporary view of the paramount importance of synaptic function: “Most of what the brain does is accomplished by synaptic transmission between neurons, and by calling upon the information ended by past transmissions across synapses. Given the importance of synaptic transmission in brain function, it should practically be a truism to say that the self is synaptic. What else could it be?”.

However, synaptologist concluded years before that the synapse alone could not account for such a sweeping interpretation. They state that “It will also become increasingly apparent that synapses account for only a subset of the entire range of interactions that exist between neurons and glia and that contribute to their functional properties in mediating behaviour. Neuroglia contribute to the processing of information by neurons through a wide range of mechanisms traditionally viewed as nonneural and nonsynaptic” [96].

Hebb [97] introduced the most influential hypothesis regarding the mechanism in which individual synaptic activity could lead to dynamic changes in groups of neurons. He proposed that “traces of memory are stored through structural modification of synaptic connections, which result in changes in synaptic efficiency and therefore, in formations of new patterns of neural activity” [94]. This is the basis of neuronal plasticity, which is “usually defined as changes which lead to an increase or decrease in the strength of synaptic signalling, associated with changes in neurotransmitter release or receptor expression” [98]. Such changes are the basis of synaptic plasticity (SP) and LTP (the increase in the strength of synaptic signaling) and long term depression (LTD) (the decrease in the strength of synaptic signaling).

Karczmar and Eccles [99] were critical of Hebb’s hypothesis. They noted that synaptic information is rarely mnemonic. Therefore, frequent synaptic activation would lead to deterioration of any memory trace. They concluded that “evidently, frequent synaptic excitation could hardly provide a satisfactory explanation of synaptic changes involved in learning” [99] (p. 57).

Recent technical advances in dating the lifetime of molecules present a significant challenge to the concept that the synapse is structurally and metabolically capable of encoding memories, which can last for days, weeks or even a lifetime. For instance, the post-synaptic density is replaced each hour [100]. Actin filaments, which are responsible for the critical change in dendritic structure during SP, are replaced every 40 s [101]. Microtubules that are important in the long range transport of molecules to and from the soma to axon terminals have a half-life of only ten minutes [94].

### 2.1. Astrocytes Control the Number, Development, Maturation, Maintenance, and Elimination of Synapses

Because astrocytes express “virtually any type of receptor found in the CNS” [23], only recently has it been possible to study their influences on synapses independent of neurons. This was made possible by the development of pure retinal ganglion cell cultures in a serum-free media [102].

The initial report utilizing this technique demonstrated that the addition of astrocyte cultured media increased the number of functional synapse sevenfold and was required for synaptic maintenance. It was concluded that “CNS synapse number can be profoundly regulated by nonneuronal signals, and raise the possibility that glia may actively participate in synaptic plasticity” [103].

The following is a summary of astrocyte-secreted molecules identified by this method to date and their effect on synapse formation and development. Several extensive reviews are available [26,31,104,105,106].

1. Thrombospondins (TSP). TSP1 and TSP2, large oligomeric extracellular matrix proteins, are necessary and sufficient to stimulate a strong increase in synapse number [107]. However, TSP-induced synapses, although ultrastructurally normal, are postsynaptically inactive due to the lack of AMPA glutamate receptors on the postsynaptic side. The α2δ-1 subunit of the voltage-dependent Ca^2+^ channel complex was identified as the neuronal receptor for TSPs [108].
  TSPs also bind to integrins, which regulate synaptic structure and function [109,110]. Additionally, TSP1 also targets the neuroligin family of synaptic adhesion proteins. “Given its ability to interact with multiple molecule types, it is possible that TSPs can not only induce synapse formation but can also modulate presynaptic and postsynaptic function” [111].
2. Glypican (Gpc) family proteins (Gpc-4 and Gpc-6) were subsequently shown to be the catalyst for the insertion of AMPA receptors into post synaptic membranes. This results in the formation of functionally active postsynaptic sites to complement the TSP induced presynaptic region [26,112].
3. Hevin and SPARC (secreted protein acidic and rich in cysteine).
  Synaptic maturation and maintenance are under the influence of these two antagonistic molecules. Astrocytes secrete hevin, which potentiates the genesis of excitatory synapses, and SPARC, which inhibits hevin-induced synaptogenesis [31,105,106].
  During development, there is a high hevin/SPARC ratio. Expression of hevin and SPARC are maintained into adulthood, indicating that hevin and SPARC might also “play changing roles in synapse maintenance and plasticity throughout life, potentially through dynamic regulation of their relative levels” [104].
  SPARC can also influence synaptic strength by interfering with integrin localization and function that normally serves to stabilize AMPA receptors at the post synaptic membrane.
4. Factors secreted by astrocytes also regulate the formation of inhibitory synapses between hippocampal neurons in vitro. They do this by modulating inhibitory postsynaptic development by stimulating neuron–neuron signaling through the neurotrophic receptor tyrosine kinase TrkB [111].

### 2.2. Elimination of Synapse and Neural Network Formation

Astrocytes label synaptic terminals with complement factor C1q on neurons that are destined for elimination during development. Microglia, as well as astrocytes, eliminate these tagged synapses by selective phagocytosis [113,114,115]. The surviving neurons and their connections constitute postnatal neuronal networks.

## 3. Gamma Oscillations

“Not only neurons but also glia and even blood vessels can contribute to the mean field measured by EEG and MEG, but in order to keep things simple, let us ignore the latter for the moment.” György Buzsáki [9].

The EEG rhythm in the gamma range (30–90 Hz) was once proposed as the “neural correlate of consciousness” that “binds” sensory information into conscious representations as well as integrate neural networks throughout the brain. This idea has been “discounted” because gamma synchrony “does not correspond with axonal firing or spikes” as originally presumed [116]. In fact, gamma synchrony, similar to all EEG waveforms, is related to surface field potentials originating from both glia and neurons [9]. Additionally, gamma synchrony is also present during non-conscious states, such as during anesthesia.

### Astrocytes Control Gamma Oscillations, Cortical UP States and Slow Wave Activity

1. Gamma Oscillations. Lee et al. [117] and associates recently demonstrate that “fast neural circuit oscillations are tightly regulated by astrocytes”. This requires the vesicular release of astrocyte neuroactive molecules known as gliotransmitters (e.g., glutamate, GABA, ATP). A transgenic mouse model, developed to inhibit vesicular release of gliotransmitters, “resulted in a marked decrease in gamma synchrony in vitro and in vivo”. Neuronal synaptic activity remained intact. A behavioral test that evaluates “the integrity of recognition memory” is abnormal. Working memory and fear conditioning remains intact. The investigators conclude that “astrocytes contribute to the fast dynamics of neural circuits that underlie normal cognitive behaviors” and are “essential contributors to information processing”.
2. Cortical UP States. Astrocytes also control cortical UP states. UP states are significantly increased neuronal synaptic firing resulting from longterm depolarization. Poskanzer and Yuste [118] show that “… activation of a single astrocyte can have significant effects on the state of large neuronal populations in a circuit, doubling the number of UP states that the circuit generates spontaneously”. Astrocyte release of glutamate is implicated in this phenomenon. The increased UP state, “working in conjunction with synaptic plasticity could create neuronal clusters that become associated with particular input signals” [49].
3. Slow Wave Activity. Astrocytes are implicated in the generation of slow wave activity (SWA) as well. Several studies have demonstrated a causal relation between astrocytic networks before it appears in neural networks during SWA. Therefore, they are critically important in long term explicit memory formation during sleep [119,120,121].

Sleep pressure increases as adenosine accumulates in the brain during waking hours. It is released as a gliotransmitter and acts through A1 adenosine receptors. The inhibition of astrocyte-specific adenosine receptors shows abnormalities in sleep homeostasis and a subsequent decline in cognitive functions. This study is the “first demonstration that a non-neuronal cell type of the brain, the astrocyte, modulates behavior and provides strong evidence of the important role of A1 receptors in the regulation of sleep homeostasis and cognitive decline associated with sleep loss” [119].

Poskanzer and Yuste [120] establish that astrocytes are important in the “slow-oscillation-dominated state shift, a neocortical rhythm characterized by synchronized neuronal firing and important for sleep and memory”. They demonstrate that “extensive synchronization of the astrocytic network precedes the spatial build-up of neuronal synchronization”. “The causal links between astrocyte activity and circuit function we uncover indicate that astrocytes are actively involved in neural circuits necessary for key brain functions such as sleep, memory and sensory processing”.

An in vivo study also demonstrates a causal link between astrocytic network synchronized activity preceding neuronal slow wave synchronization. “The earlier extensive recruitment of astrocytes in the synchronized activity is reinforced by the observation that neurons surrounded by active astrocytes are more likely to join SWA, suggesting causality”. Interestingly, the blockade of gap junctions in the AS or astrocytic calcium transients, reduced the SWA seen in both astrocytes and neurons. “These in vivo findings conclusively suggest a causal role of the astrocytic syncytium in slow wave activity” [121].

## 4. Cortical Minicolumns

“It remains true that nothing is known about the physiological correlates (if, indeed any exist) of the minicolumn”. Horton and Adams [122].

The discovery of minicolumns “crystalized conceptually the idea that the single neuron was not only the anatomical and functional unit of the brain but also its perceptual unit” [123]. Mountcastle [124] proposed the cortical minicolumn as the basic functional unit of the mammalian neocortex 70 years ago. These are “cells in any vertical cluster that share the same tuning for any given receptive field attribute”.

Each minicolumn contains between 80–100 neurons and is separated by glial septa. A defining characteristic is that they are modality specific. For example, only single angles of orientation cues elicit responses within individual minicolumns. Therefore, such structures are autonomous and segregated.

### 4.1. Radial Glial Cells Are the Precursors of Minicolumns

The structure of cortical minicolumns is genetically predetermined by RGCs. In fact, they are the mature cells of the “ontogenic columns” of neural precursor cells [124,125]. The radial unit hypothesis postulates that neurons that form the minicolumn networks of the neocortex “would be patterned by trajectories of the radial glia guiding fiber” [126].

During development, future neurons are guided along fibers of RGC’s that extend from the ventricle upward to the surface of the neocortex by radial migration. The neurons form the “radial unit”. These vertical minicolumns of neurons originate from the same RGC. In adults, cortical minicolumns are also known as modules. In fact, the terms radial columns and modules are “interchangeable” [127].

Remarkably, the size of minicolumns is preserved in brain evolution. Their size remains approximately the same even in primates with much larger brains than other mammals. Bugbee and Goldman-Rakic [85] proposed that larger brains evolve by simply adding new functional minicolumns.

Mountcastle notes the major problem in extending minicolumns to general cognitive constructs: “How the patterns of neural activity involved in a sensory discrimination or categorization, distributed as they are in wide areas of the brain, are unified into perceptual wholes, and how they flow through to conscious experience, remain among the great enigmas in brain science” [124].

This enigma is referred to as the “binding problem”. It was first elaborated over a century ago by William James who emphasized “there is no cell or group of cells in the brain of such anatomical or functional preeminence as to appear to be the keystone or center of gravity of the whole system” [128]. As proposed earlier, the AS incorporates the “group of cells” in the brain that serves this function.

### 4.2. Astrocytes, Cortical Columns, and Singularities

Similar to cortical minicolumn neurons, astrocytes in the visual cortex also respond to spatial and orientation cues in vivo, with astrocytes often more responsive [129]. As many as ten astrocytes may respond to an individual orientation cue indicating that they are organized into “functional domains” [130]. Most interesting is that two astrocytes that are tens of microns apart may respond to completely different orientation cues. The close approximation of the astrocytes would indicate that they are likely in the same minicolumn, but responsive to more than one orientation cue, in contrast to their neuronal counterparts [15].

In addition to minicolumns, in vivo studies of the receptive fields across the visual cortex of ferrets, known as pinwheels or singularities, indicate that there is a perfect overlay of neurons and astrocytes. Furthermore, astrocytes are more responsive than neurons, particularly in the pinwheel center, the area of confluence of different orientation patterns [129]. Importantly, these patterns are not related to presynaptic neuronal activation or other neural responses. “This indicates that astrocytes are directly processing visual information rather than simply responding to neural signalling” [15].

### 4.3. Neuronal Empirical Contradictions to the Cortical Minicolumn Concept

Horton and Adams note: “The cortical column also has an ‘Achilles heel’. They are present in some species and absent in others. Furthermore, they may be present or absent in individuals of the same species. For instance, orientation columns in the striate cortex are found in, among others, apes, monkeys, tree shrews, sheep and ferrets. However, they are absent in rats, mice and rabbits… Ocular dominance columns are also absent in these species… Because of the absence of cortical columns in certain species, it has been speculated that they are striking but inconstantly expressed anatomical feature that has no obvious relationship to function” [122].

## 5. Radial Glial Cells and Astrocytes Are Targets of Human-Specific Genes that Increase Brain Size, Metabolism, Adult Synaptic Maintenance, Gyrification, and Neoteny

### 5.1. CA2 (Carbonic Anhydrase II) Gene and Increased Human Brain Metabolism

CA2, the gene responsible for carbonic anhydrase is up regulated in human brains. It is localized in astrocytes and necessary for the transport of lactate from these cells to neurons [131]. As reviewed earlier, lactate is essential for neuronal survival because fastidious neurons are missing two essential enzymes in the tricarboxylic cycle. Interestingly, this is unusual from an evolutionary perspective. Larger brains generally have a decrease in metabolic rates than species with smaller brains [87].

### 5.2. TSP2 and TSP4 Genes and Synaptic Organization and Plasticity in Adult Human Brains

TSP1 and TSP2 have been mentioned previously because of their ability to induce synaptogenesis during development. They generally decline prior to birth. However, TSP 2 and TSP 4 are “upregulated in human brain evolution” [132]. They have a six-fold greater expression of their mRNA and associated proteins in adult humans compared to chimpanzees. Most interestingly, they are primarily located in the forebrain of humans, but not in other areas of the CNS [131].

This was the first study to demonstrate changes in human gene expression in a specific area of the neocortex. Additionally, TSP4 antibodies label amyloid plaques in patients with Alzheimer’s disease. “Increased expression of thrombospondins in human brain evolution could result in changes in synaptic organization and plasticity, and contribute to the distinctive cognitive abilities of humans, as well as to our unique vulnerability to neurodegenerative disease” [132].

### 5.3. ARHGAP11B Gene and Increased Size and Gyrification of the Human Brain

ARHGAP11B has the “highest degree of radial glia-specific expression” in the human neocortex. It is the product of a partial duplication of the ARHGAP11A gene that occurred after the separation of humans and chimpanzees approximately five million years ago. It “promotes basal progenitor generation and self-renewal and can increase cortical plate area and induce gyrification” [133]. Interestingly, the gene is also present in Neanderthal and Denisovans DNA, extinct large brain hominids.

Most astonishing is the recent finding that ARHGAP11B obtained its ability to induce increased divisions of basal progenitor cells and the subsequent increased expansion of the human brain only when a C to G base substitution occurred in ARHGAP11B. Therefore, “the ability of ARHGAP11B likely arose more recently from a change that is tiny on a genomic scale but substantial in the functional and evolutionary consequences” [134].

### 5.4. SRGAP2C Paralog of the Ancestral SRGAP2 Gene and Neoteny

SRGAP2C occurred by incomplete segmental duplication of the SRGAP2 gene [135,136]. The SRGAP2C paralog induced neoteny by interfering with the timing of dendritic spine maturation function of SRGAP2. This occurred 2–3 million years ago “corresponding to the transition from Australopithecus to Homo and the beginning of neocortex expansion” [135]. It is also present in Neanderthal DNA [136].

At birth, the ancestral SRGAP2 slows migration of neurons and promotes the maturation of dendritic spines. However, the novel paralog has the opposite effect by significantly slowing spine maturation. Ultimately, human dendritic spines become longer, more numerous and more complex. This contributes to the “high level of synaptic densities in the human cortical neuropil compared to other primates and rodents” [87].

Therefore, there is an extended period of postnatal brain development in which the brain is not fully formed until approximately the age of 20 or older. Ultimately, this accounts for human postnatal intellectual development and increased brain size, probably in conjunction with ARHGAP11B.

## 6. Discussion

The basis of all cognitive functions, including learning, memory, and consciousness, remains elusive despite the accumulation of massive empirical data for over a century under the influence of the tenets of the Neuron Doctrine and the assumption that the synapse is the locus of all cognition.

Seventy years have elapsed since Hebb’s hypothesis regarding the critical nature of LTP or LTD culminating ultimately in memory formation. This is “assumed”, but not proven. Furthermore, the proposed functional importance of cortical minicolumns and cortical electrical activity as the ultimate locus of conscious expression, memory storage and recall, as well as learning, are no longer tenable.

The failure of all such proposals has ultimately led to further neurocentric hypotheses that are actually non-scientific and damaging to scientific inquiry. It must be stressed that the Neuron Doctrine is dogma and, as such, has not been unequivocally validated. The elevation of this dogma to doctrine, which is more a policy or religious term than a scientific one, is unfortunate. This has led ultimately to the teleological idea that consciousness, the fundamental basis of memory and learning, is an “emergent” phenomenon that occurs as intelligence increases with increased brain size in humans [137,138,139].

The concept of “emergence” is not subject to empirical scrutiny. It has been adopted by philosophers and religious pundits to “prove” that there is an ephemeral state or “director” that is beyond scientific inquiry. Unfortunately, it has also led many neuroscientists to propose that a reductionist explanation can never provide insight into cognitive functions. Such alarming and pessimistic statements are directly linked to inflexible neurocentric interpretations.

Every biologist learns very early that teleological statements of this kind are to be rigorously avoided. To do so leads to mystical and anthropomorphic proposals that are distinctly at variance with the Scientific Method and are inconsistent with the Theory of Evolution.

In retrospect, it is easy to see how the neuron became the dominant cell of focus. Golgi’s silver impregnation technique exclusively stained neurons. Action potentials were easily studied by oscilloscopes, particularly in invertebrate models such as the squid giant axon. All of the basic neurocentric tenets and proposals, particularly the Neuron Doctrine, are based on innumerable fallacies when examined in light of contemporary empirical evidence, particularly knowledge gained over the past three decades by gliobiologists.

The accumulating evidence that glia, particularly astrocytes, are involved in all facets of cognitive processes has been possible with the development of ingenious technical accomplishments. This includes the ability to distinguish glial activity from neuronal activity on a millisecond time frame at the level of an individual synapse.

The astonishing empirical evidence of the importance of glia in higher brain functions should be greeted with enthusiasm by all neuroscientists. It opens prospects for further investigations in the quest for explanations of all cognitive realms. It gives a new direction to recover from the doldrums of neurological investigations into memory, consciousness, and learning that have been apparent for decades.
